# Trop2 binding IGF2R induces gefitinib resistance in NSCLC by remodeling the tumor microenvironment

**DOI:** 10.7150/jca.57711

**Published:** 2021-07-03

**Authors:** Xia Sun, Lizhou Jia, Tengqi Wang, Yulian Zhang, Wei Zhao, Xiangcheng Wang, Hao Chen

**Affiliations:** 1 Emergency Center, Bayannur Hospital, Bayannur, Inner Mongolia, 015000, China.; 2 Department of Pathology, Wannan Medical College, Wuhu, Anhui, 241002, China.; 3 Cancer Center, Bayannur Hospital, Inner Mongolia, 015000, China.; 4 Department of Pathology, Nanjing First Hospital, Nanjing, Jiangsu, 211166, China.; 5 Department of nuclear medicine, The Affiliated Hospital of Inner Mongolia Medical University, Hohhot, 010050, China.; 6 Key Laboratory of Inner Mongolia Autonomous Region Molecular Imaging, Inner Mongolia Medical University, Hohhot, 010050, China.; 7 Faculty of medical science, Jinan University, Guangzhou, Guangdong, 510632, China.

**Keywords:** NSCLC, gefitinib, drug resistance, Trop2, IGF2R, TME

## Abstract

Gefitinib has shown good efficacy in treating recurrent or advanced non-small cell lung cancer (NSCLC), but the drug resistance remains a clinical challenge in medical oncology. In addition, the complex interaction between tumor cells and heterogeneous stromal cells in the adjacent tumor microenvironment (TME) is also an important contributor to drug resistance. So, it is very necessary to detect the related target genes before and after gefitinib treatment dynamically. In this study, the relationship between Trop2 and gefitinib resistance in NSCLC was investigated, and the underlying mechanism was explored. Results showed that Trop2 was associated with EGFR gene mutation and drug resistance in clinical tissues. Trop2 was confirmed to induce gefitinib resistance in NSCLC, and Trop2 binding IGF2R promoted the IGF2-IGF1R-Akt axis to enhance gefitinib resistance and remodeling the TME in NSCLC. Notably, silencing of Trop2 in cancer cells combined with IGF1R inhibitor significantly decreased the proliferation of tumor cells and reshaped the NSCLC TME *in vivo* and *in vitro*, including the recruitment of macrophages. These findings deepened the understanding of the function of Trop2 and the involved mechanisms of gefitinib resistance, and may provide new molecular targets for NSCLC with gefitinib resistance.

## Introduction

The occurrence and development of tumors depend not only on gene mutations, but also on the heterogeneity of the tumor microenvironment (TME) 1-3. The dynamic changes of TME heterogeneity depend on immune cells, immune mediators and their gene and protein profiles 4. Tumor cells promote their uncontrolled proliferation and metastasis through remodeling the TME 5. The immune network in the TME, which contains both anti-tumor and pro-tumor factors, determines the initiation, development and outcome of the tumor. The interactions among genes, proteins and cells in the TME have become the targets of diagnosis and treatment strategies for cancer. Targeted therapy has the long-term growth potential on the basis of TME heterogeneity 6,7.

Tyrosine kinase inhibitors (TKIs) have been used at the forefront of non-small cell lung cancer (NSCLC) therapy in recent years, but some patients acquire drug resistance to EGFR-TKIs such as gefitinib 8,9. Therefore, drug resistance is still a difficult problem in clinical treatment. Based on the challenges with gefitinib resistance and its unknown mechanisms, the detection of target genes before and after gefitinib treatment for individualization and the accuracy of clinical treatment need to be researched 10,11.

The insulin receptor family belong to the receptor tyrosine kinases (RTK) subfamily, members of which include insulin receptor (IR), insulin-like growth factor receptor (IGFR) and insulin receptor-related receptor (IRR) 12,13. When binding to their respective ligands (such as IGF-1 or IGF-2), members of the insulin receptor family activate intracellular tyrosine kinases and initiate intracellular signal transduction through a series of structural and conformational changes, which play important physiological roles in organisms 14,15. Insulin-like growth factor-1 receptor (IGF1R) has been regarded as one of the most promising targets for the treatment of NSCLC, and many treatments targeting IGF1R are applied in clinical trials. However, the overall response rate of the IGF1R inhibitor treatment is not satisfactory 16.

Human trophoblast surface antigen 2 (human trophoblast cell surface antigen 2, TACSTD2/Trop2/M1S1/GA733/1) is a transmembrane protein encoded by the TACSTD2 gene 17,18. Trop2 is widely expressed on the surface of many kinds of epithelial cell carcinoma but rarely expressed in non-tumorous human tissues 19. Previous studies reported that Trop2 is lower expressed in lung adenocarcinoma due to epigenetic inactivation and inhibition of IGF1 signaling pathway, and the deletion of Trop2 in squamous cell carcinoma promotes tumorigenesis and the transformation from epithelium to mesenchymal cells 20,21. Howerver, the specific role of Trop2 in lung cancer remains unknown.

In this study, we demonstrated that Trop2 binding IGF2R increased the IGF2-IGF1R-Akt axis to promote EGFR-TKI resistance and remodeling the TME in NSCLC. We found that Trop2 physically interacted with IGF2R and resulted in cytokine production, TME remodeling, recruitment of infiltrated macrophages, and the proliferation of cancer cells. Moreover, IGF signaling activated by Trop2 promoted NSCLC resistance to gefitinib *in vivo* and *in vitro*. Downregulation of Trop2 in the xenograft model with IGF1R inhibitor significantly decreased the proliferation of tumor cells and remodelled TME.

## Material and methods

### Patients and IHC staining

A total of 164 NSCLC and 32 paracancerous tissues were collected from Nanjing First Hospital and Bayannur Hospital (Jan.2015-Dec.2016). The clinicopathological information of the patients included gender, age, histological type, differentiation, TNM stage, tumor size, lymph node metastasis, distant metastasis, EGFR mutation and the effectiveness of gefitinib (83 of the 164 patients were treated with gefitinib). The data of EGFR gene Mutation (EGFR Exon18-21) were detected by ARMS-PCR. This experiment was approved by the ethics committee of Nanjing First Hospital and Bayannur Hospital.

Anti-Trop2 antibody (Abcam, ab214488, Rabbit, 1:100) was used to detect the protein expression in the tissue samples according to the manufacturer's instructions. The protein expression was scored using the semi-quantitative H-score method, and cut off point was set to 130 22.

### Cell lines and reagents

Wi38, THP-1 and PC-9 cells were purchased from Shanghai FuHeng Biology co., LTD (China). Cells were cultured as previously described 23. The cells were not contaminated by mycoplasma. Gefitinib-resistant PC-9/GR cells were constructed through exposing PC-9 to gradient increased concentration of gefitinib for 10 months. 293T and 293 cells were donated by Key Laboratory of Antibody Technique of National Health Commission, Nanjing Medical University 17,18. Linsitinib (IGF1R inhibitor) was purchased from MedChemExpress co., LTD (USA) and gefitinib was purchased from Astrazeneca LTD (England).

### Plasmids, lentivirus and cell transfection

Transfection with expression plasmid was performed using Lipofectamine 2000 (Invitrogen), and the transfected method could be seen in manufacturer's protocol (GeneCopoeia, China). ShRNA lentivirus plasmids targeting Trop2 or IGF2R were synthesized by Guangzhou GeneCopoeia Company. OE/shTrop2 plasmid were preserved in our Laboratory.

### Western blotting and co-immunoprecipitation assay

Proteins from the extracts of cells were detected by western bloting according to standard protocols. Primary antibodies used in the experiment as followed: anti-Trop2 antibody (Abcam, ab214488, Rabbit, 1:1000), anti-GAPDH antibody (Abcam, ab181602, Rabbit, 1:2000), anti-IGF2R antibody (Proteintech, 20253-1-AP, Rabbit, 1:1000), anti-IGF2 antibody (Abcam, ab170304, Rabbit, 1:1000), anti-*p*-IGF1R antibody (Abcam, ab39395, 1:1000), anti-Akt antibody (CST, 4685, Rabbit, 1:1000), anti-*p*-Akt antibody (CST, 4060, Rabbit, 1:1000).

Cells transfected with plasmid for 48 h were lysed in RIPA buffer containing a protease inhibitor cocktail (Sigma-Aldrich, USA). For immunoprecipitation assays, Dynabeads® beads were placed in a tube along with 10 μg mouse anti-human Flag antibody or control IgG and 200 μl PBST, and samples were incubated for 10 min at room temperature. The beads were washed with 200 μl PBST and 500 μl cell lysate was added. Then samples were incubated at room temperature for 10 min or 4 °C overnight. The Dynabeads®-Ab-Ag complex was washed three times with 200 μl washing buffer and transferred to a new tube. The supernatant was removed, and 20 μl elution buffer and 10 μl premixed NuPAGE® LDS sample buffer and NuPAGE® sample reductant were added. The samples were incubated at 70 °C for 10 min, and then the supernatants were examined by western blotting.

### Cell viability, migration, co-culture assays

Viability of NSCLC cell lines in gefitinib was detected by CCK-8. Transduced cells were transferred into medium, and exposed to the test gefitinib (0-0.1 μM) for 72 h. The migration of NSCLC cell lines was detected by transwell assay using Millipore filters. PC-9/GR cells were transduced plasmid and exposed to media for 24 h, and the cells that migrated into the lower chambers were stained by crystal violet. Transwells (0.4 μm pore size, Corning) were used in co-culturing experiment for different time, and then the cells were harvested.

### Mass Spectrometry

Dynabeads® magnetic beads, mouse anti-human Flag antibody, control IgG, OE-Trop2-293T cell lysates were cultured, and the compound was sent to protein spectrum. After elution, the compound was analyzed by capillary high performance liquid chromatography (HPLC). MaxQuant 1.5.2.8 software was used to analyze the data, and 26 trop2-interacting proteins were obtained.

### Mouse model

NOD/SCID mice were purchased from Beijing Vital River Laboratory Animal Technology. For orthotopic tumor models, PC-9/GR shNC and PC-9/GR shTrop2 cells (1 × 10^6^ cells in 100 μl media) were injected in 4-5 weeks old NOD/SCID mice (n = 5 per group) at chest sites respectively. Gefitinib was treated by the oral administration (50 mg/kg). When the solid tumor reached a volume of 100 mm^3^, mice were treated with the IGF1 inhibitor linsitinib (10 mg/kg, i.p. once a week) or PBS. Tumor volume was calculated as follows: V = 1/2AB^2^, where A represents the longest diameter, and B represents the vertical diameter. Mice were euthanized when tumors reached >10% of body weight or mice became moribund. The tumors were harvested for immunohistochemistry and H&E Staining.

### Statistical analysis

The SPSS18.0 statistical software package (SPSS Inc., Chicago, IL, USA) was used for general statistical analysis. Pearson's χ^2^ test was used to compare expression level of target gene in tissue samples and the association with clinical pathological parameters. NSCLC patients' OS was evaluated with Kaplan-Meier and Log-rank method. Univariable and multivariable Cox proportional hazard regression models were used to analyze independent prognostic factors. The differences between two groups were analyzed with unpaired Student's *t*-test. *P* < 0.05 was considered statistically significant.

## Results

### Trop2 was aberrantly expressed in EGFR mutant NSCLC tissue samples and associated with gefitinib resistance

**Figure 1:**
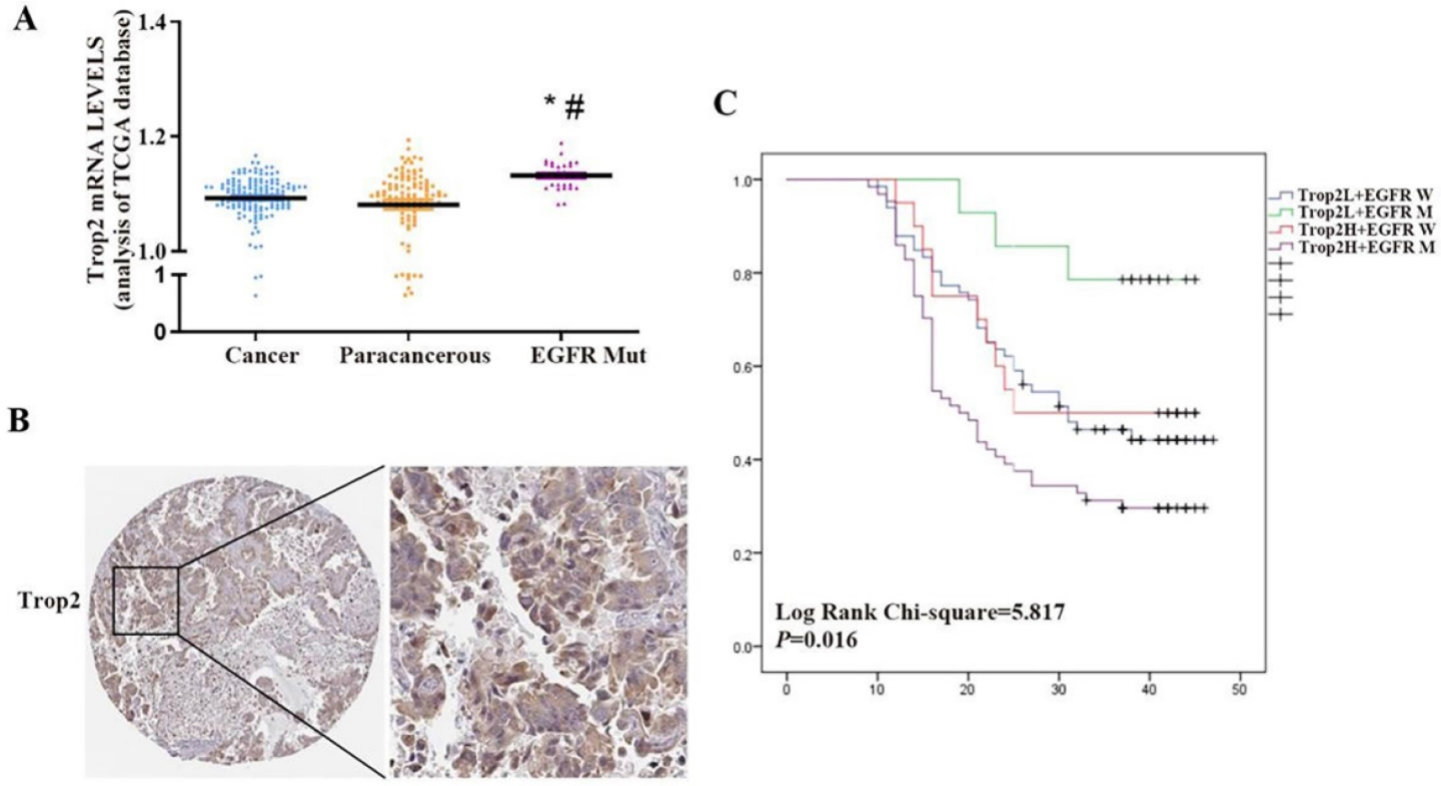
** Trop2 was aberrantly expressed in NSCLC tissue samples with EGFR mutation and associated with poor survival prognosis. (A)** The Cancer Genome Atlas (TCGA) measured the expression difference of Trop2 in NSCLC cancer, paracancerous and EGFR mutated (EGFR Mut) tumor tissues, *P<0.05, means compared with cancer group, ^#^*P*<0.05, means compared with paracancerous group. **(B)** Representative image of higher Trop2 expression in NSCLC tissue sample by IHC. **(C)** OS curves for patients with low or no Trop2 expression and EGFR wild (blue line), with low or no Trop2 expression and EGFR mutation (green line), with high Trop2 expression and EGFR wild (red line), with high Trop2 expression and EGFR mutation (purple line).

Trop2 is widely expressed in many kinds of epithelial cell carcinoma. However, some reports suggested that Trop2 is expressed at low levels in lung cancer. Using the publicly available gene expression database The Cancer Genome Atlas (TCGA), we found that there was no significant difference in the expression level of Trop2 between NSCLC and paracancerous tissues, but the expression level of Trop2 in NSCLC tissues with EGFR mutation was higher than that in paracancerous tissues (Fig. 1A). We performed immunohistochemistry on 164 NSCLC and 32 paracancerous tissues, and found that the expression level of Trop2 in lung cancer tissues was not significantly different from that in paracancerous tissues (Table S1). Analysis of the clinicopathological data of cases revealed that the expression of Trop2 was related to EGFR gene mutation. The high expression rate of Trop2 in NSCLC tissues with EGFR mutation was 82.10% (64/78), which was higher than that in tissues without EGFR mutation (23.30%, 20/86) (Table 1) (Fig. 1B). Meanwhile, we also found that NSCLC patients with high Trop2 expression developed drug resistance earlier in the course of taking gefitinib (Table 1). Further analysis showed that NSCLC patients with Trop2 high expression and EGFR mutation were significantly associated with poor overall survival (Fig. 1C).

### Trop2 induced gefitinib resistance in NSCLC

**Figure 2:**
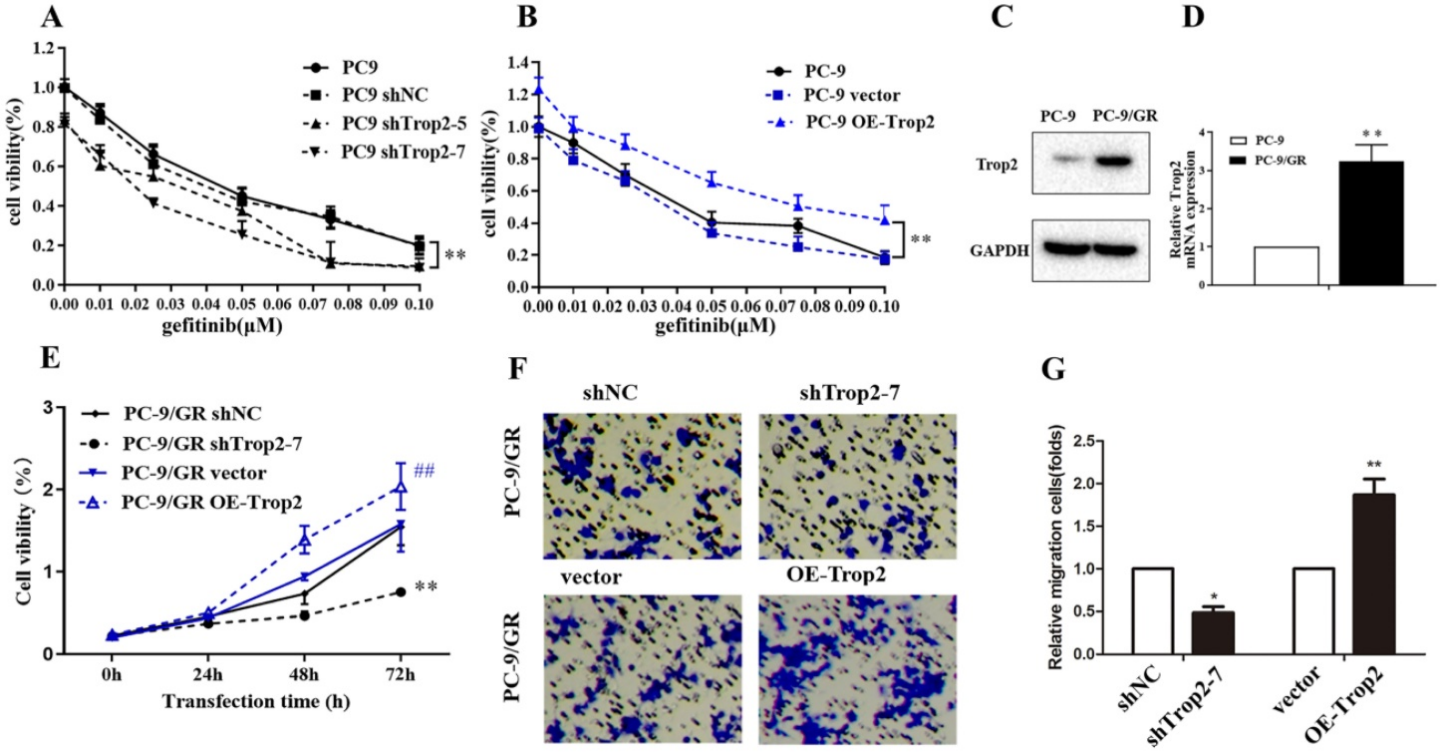
** Trop2 induced gefitinib resistance in NSCLC. (A-B)** The cell viability of NSCLC cell lines with gefitinib treatment, which were been knock down (A) or over-expression (B) of Trop2. Mean ± SD, ** P < 0.01. **(C-D)** Trop2 expression was tested in NSCLC cell lines (PC-9) and gefitinib drug-resistance cell lines (PC-9/GR) through western blotting (C) and qRT-PCR (D), Mean ± SD, **P<0.01. **(E)** Cell viability was detected in PC-9/GR cells, harboring control (shNC), silencing of Trop2 (shTrop2-7), vector, or over-expression of Trop2 (OE-Trop2) at 0 h, 24 h, 48 h and 72 h with gefitinib treatment. Mean ± SD, **P<0.01, means compared with shNC group, *^##^P*<0.01, means compared with vector group. **(F-G)** Migration assay of PC-9/GR cells transduced with shTrop2-7 or OE-Trop2 plasmid companied with gefitinib treatment at 24 h. Representative images of cell migration were shown (F), and (G) is the statistical graph of (F), Mean ± SD, *P<0.05, **P<0.01.

Trop2 in NSCLC cell lines was knock-downed or over-expressed as shown in Fig. S1A-B. Then the cell viability of these cells treated with gefitinib were examined. The results indicated Trop2 increased cell lines resistance to gefitinib (Fig. 2A-B). Next, we detected Trop2 expression in NSCLC cell lines (PC-9) and cell lines resistant to gefitinib (PC-9/GR). We found that the expression of Trop2 was at higher levels in gefitinib-resistant cell lines, compared with parental cell lines (Fig. 2C-D). To analyze the role of Trop2 in PC-9/GR cells, we knocked down (shTrop2) and over-express Trop2 (OE-Trop2) in PC-9/GR (Fig. S1C-D). The results indicated that knock-down of Trop2 markedly inhibited the ability of cell proliferation and migration, and over-expression of Trop2 significantly facilitated the ability of cell proliferation and migration with gefitinib treatment (Fig. 2E-G). These results indicated that Trop2 induced gefitinib resistance in NSCLC.

### Trop2 interacted with IGF2R

**Figure 3:**
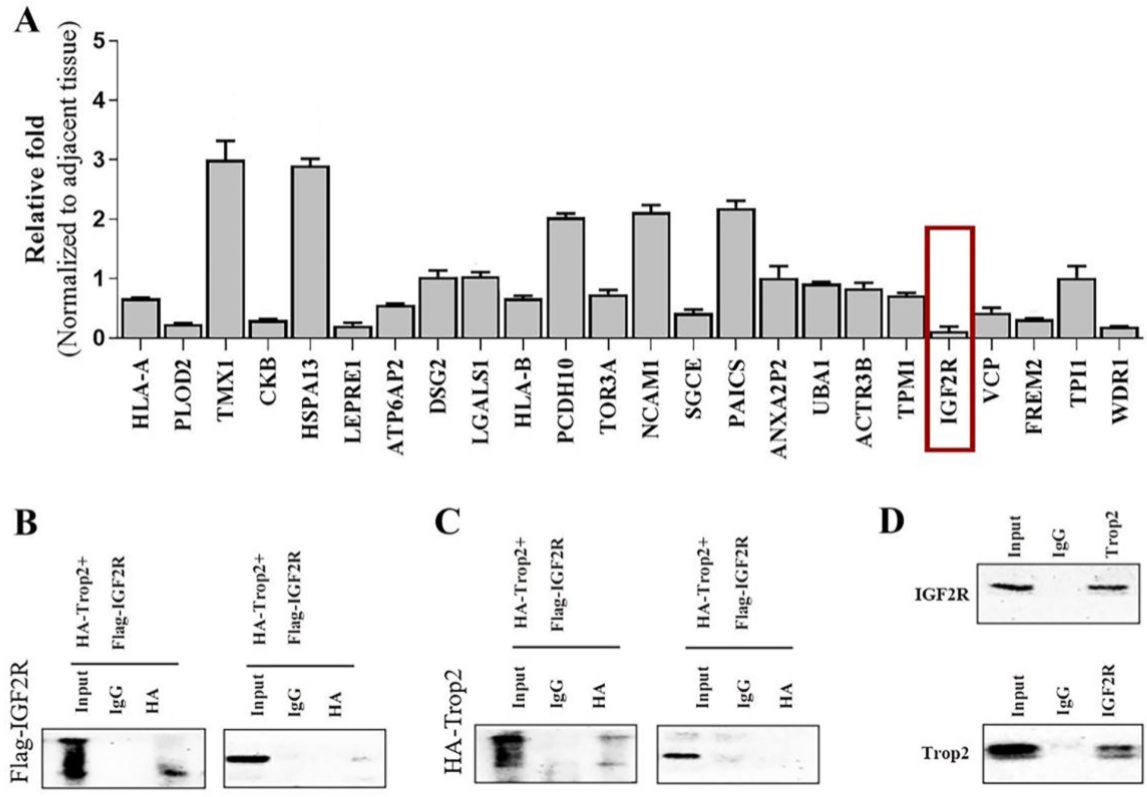
** Trop2 physically bound with IGF2R. (A)** Trop2 was over-expressed in PC-9/GR cells and the mRNA levels of the interacting proteins were detected by qRT-PCR. **(B)** 293T cells were transfected with HA-Trop2 or control plasmid HA-PCMV and Flag-IGF2R plasmid, and the cell lysates were immunoprecipitated with anti-flag antibody. **(C)** 293T cells con-transfected with Flag-IGF2R or control plasmid Flag-pcDNA3.1 and HA-Trop2 were coimmunoprecipitated by immunobloting with anti-HA-antiboby. **(D)** PC-9/GR cell lysates were immunoprecipitated with anti-Trop2 antibody and the coimmunoprecipitated IGF2R was detected using anti-IGF2R antibody.

We generated the over-expression plasmid of Trop2 and transfected the plasmid into 293T cells. Using spectrometry, the Trop2-interacting proteins were obtained (data not shown). We then used qRT-PCR to examine the mRNA expression levels of the related proteins in NSCLC cells when over-expressing shTrop2. The results revealed that the mRNA level of IGF2R showed an obvious reduction (Fig. 3A). IGF2R may be the potential protein which is inhibited by Trop2 and we therefore focused on IGF2R in the follow-up experiments.

To more closely examine the Trop2 and IGF2R interaction, we transfected Flag-IGF2R plasmid and HA-Trop2 plasmid in 293T cells. As shown in Fig. 3B-C, we observed physical interactions of Trop2 and IGF2R. Pull-down assay also confirmed the interaction of the two proteins in PC-9/GR cells (Fig. 3D).

### Trop2 bound with IGF2R to activate the IGF2-IGF1R-Akt axis in NSCLC gefitinib resistance

**Figure 4:**
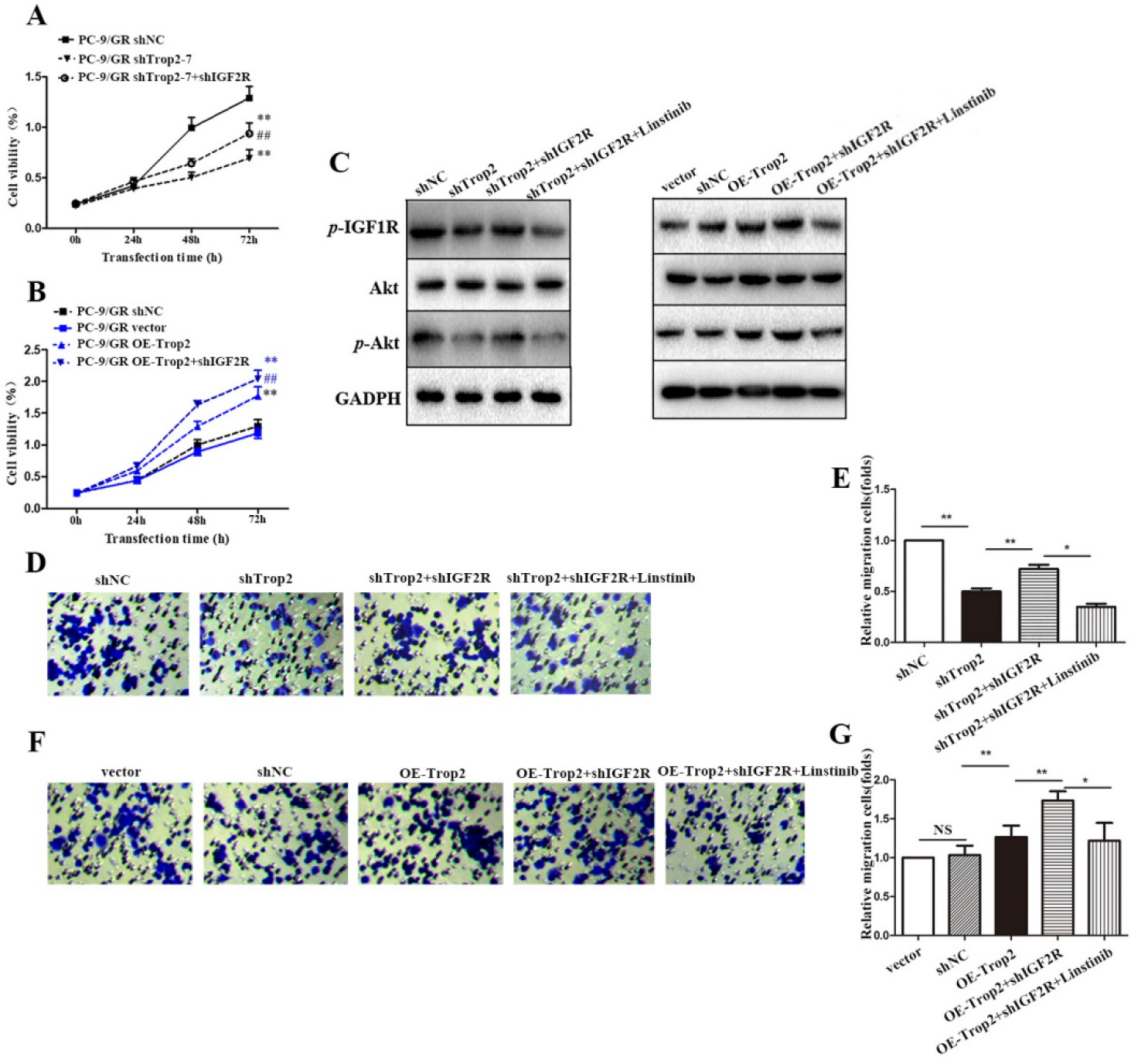
** Trop2 bound with IGF2R to activate IGF2-IGF1R-Akt axis in NSCLC gefitinib resistance. (A)** Cells were transfected with shNC, shTrop2, and/or shIGF2R plasmids respectively with gefitinib treatment for 72 h to detect the proliferation ability. Mean ± SD, **P<0.01, means compared with shNC group, *^##^P*<0.01, means compared with PC-9/GR shTrop2 group. **(B)** Cells were transfected with vector, OE-Trop2, and/or shIGF2R plasmids respectively for 72 h to detect the proliferation ability. Mean ± SD, **P<0.01, means compared with vector group, *^##^P*<0.01, means compared with PC-9/GR OE-Trop2 group. **(C)** PC-9/GR was knocked down or over-expressed with shIGF2R or linstinib for 24 h, Western blotting was used detect the protein level of *p*-IGF1R, Akt and *p*-Akt. **(D-G)** The migration of cells were detected through migration assay, and the representative images of cell migration were shown in (D) and (F), (E) is the statistical graph of (D), and (G) is the statistical graph of (F), Mean ± SD, *P<0.05, **P<0.01.

To further explore the mechanism of Trop2 in gefitinib NSCLC resistance, we generated a shIGF2R plasmid, and then co-transfected shTrop2 and shIGF2R plasmid into PC-9/GR cells. Transfection of shIGF2R plasmid partly restored the proliferation of OE-Trop2 PC-9/GR cells (Fig. 4A-B). We also examined the protein and mRNA expression levels of IGF axis proteins by western blot. Trop2 increased the expression levels of *p*-IGF1R and Akt through binding IGF2R. However, linsitinib, an IGF1R inhibitor, abolished IGF1R expression and reversed the changes in the expression of IGF axis proteins (Fig. 4C). The similar results were also verified through migration assays, and we found IGF1R inhibition decreased the migration of cells (Fig. 4D-G).

### Trop2 activated IGF2 in combination with IGF2R to reshape the TME

**Figure 5:**
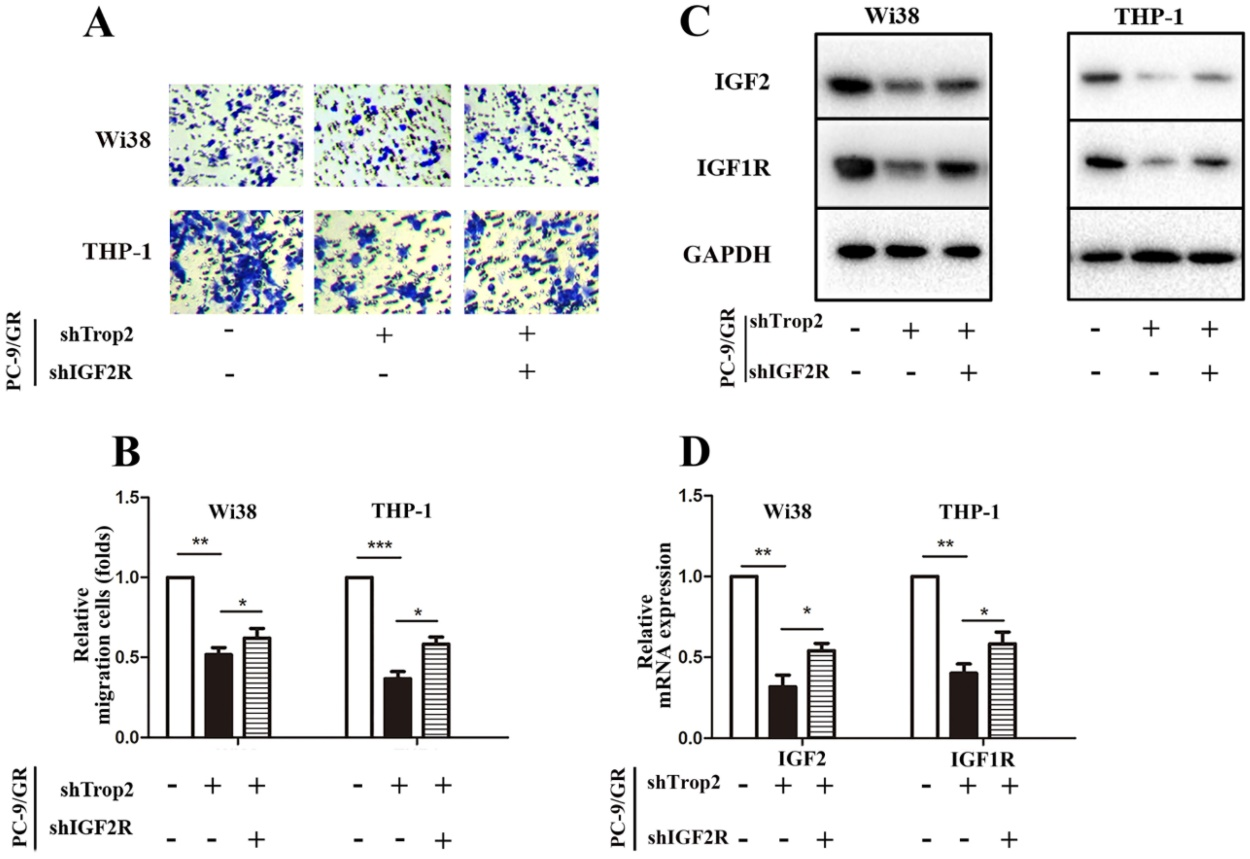
** Trop2 activated IGF2 to reshape tumor microenvironment in NSCLC gefitinib resistance. (A-B)** Co-culture system through migration assay, Wi38 or THP-1 cells were added in upper chamber, and PC-9/GR, PC-9/GR shTrop2 or PC-9/GR shTrop2+shIGF2R were added in lower chamber with gefitinib treatment for 24 h or 48 h; (A) Representative images of the cell migration were shown; (B) is the statistical graph of (A). **(C)** The protein levels of IGF2 and IGF1R were detected at 48 h in Wi38 and THP-1 cells. (D-E) The mRNA levels of IGF2 and IGF1R at 24 h in Wi38 and THP-1 cells. Mean ± SD, *P<0.05, **P<0.01, ***P<0.001.

As IGF1R inhibitor fails to totally reverse gefitinib resistance in NSCLC, we speculated that the complex TME in NSCLC also contributes to the occurrence of drug resistance. Drug-resistant cancer cells can recruit macrophages and fibroblasts, and these cells then recruit a large amount of vascular endothelial cells. Therefore, we examined whether Trop2 binding IGF2R functions in drug resistance within the crosstalk between infiltrating stromal cells and gefitinib resistant cancer cells. We performed a transwell migration and co-culture assay. To further investigate the molecular mechanism of Trop2 in TME, we added human embryonic lung cells Wi38 or mononuclear macrophage THP-1 cells in the upper chamber and added PC-9/GR, PC-9/GR shTrop2 or PC-9/GR shTrop2+shIGF2R cells in the lower chamber. We found that the presence of PC-9/GR cells resulted in increased the migration of Wi38 and THP-1 cells, and knockdown of Trop2 decreased the migration of Wi38 and THP-1 cells (Fig. 5A-B). To detect whether the IGF axis functions in infiltrating stromal cell recruitment, we carried out qRT-PCR and western blot assay. IGF2 was higher in Wi38 and THP-1 cells that were co-cultured with PC-9/GR cells, while the lower expression of IGF2 was detected in the cells co-cultured with shTrop2-PC-9/GR cells and was reversed by shIGF2R. The same changes of IGF-1R expression were observed (Fig. 5C-D).

### Inhibition of Trop2 in NSCLC reduced gefitinib resistance combining with linstinib in mice

**Figure 6:**
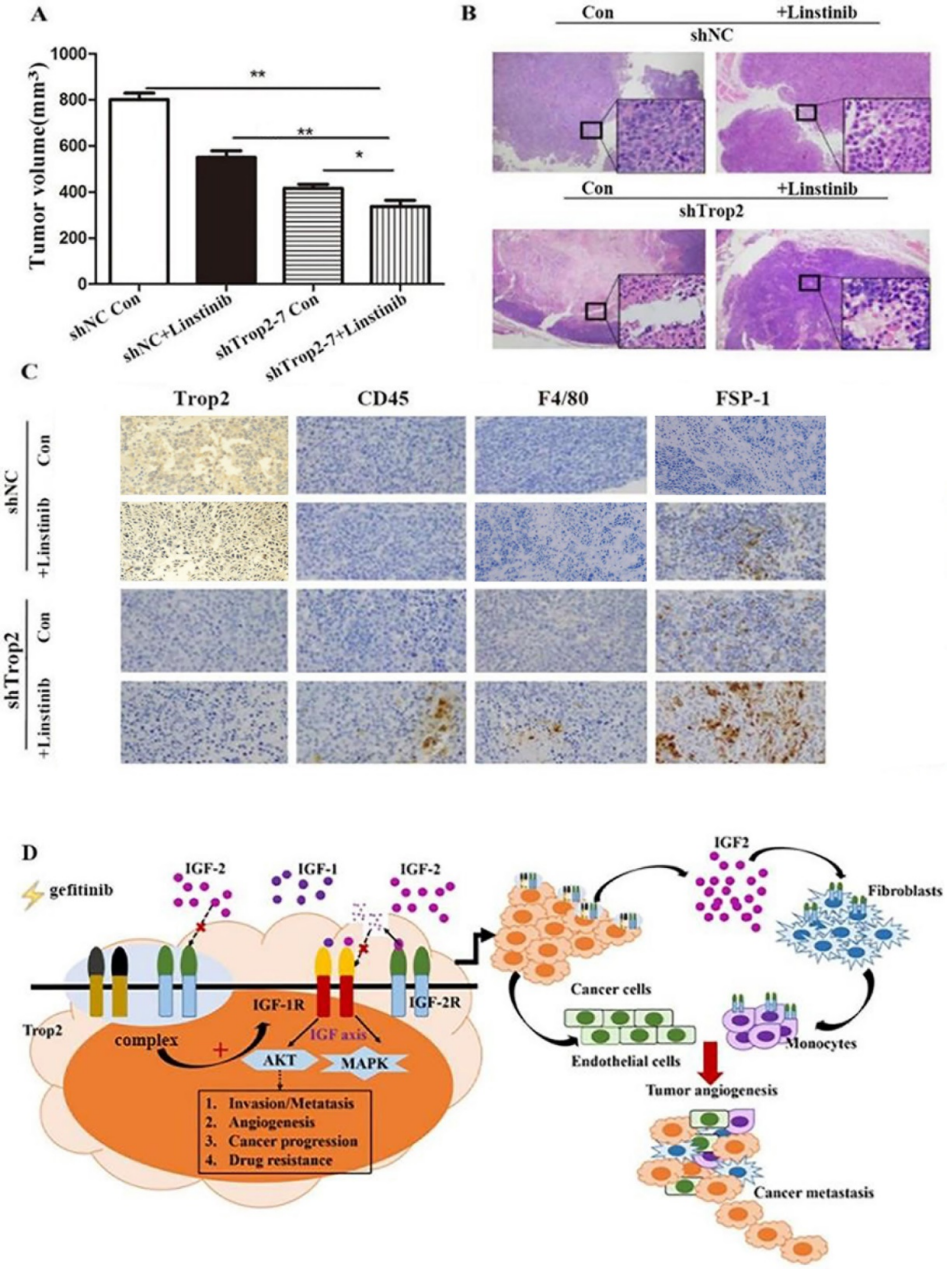
** Knockdown of Trop2 reduced gefitinib drug resistance combining with inhibiting IGF1R* in vivo.* (A)** Nude mice bearing PC-9/GR shNC or shTrop2 cell lines xenograft tumors were treated with or without linstinib, companied with gefitinib oral administration. At the end of experiment, representative tumors were harvested, every animals were monitored for the change of tumors volume, Mean ± SD, *p < 0.05, **p < 0.01. **(B)** At the end, H&E staining of the tumor samples from mice were performed (amplification ×4, inside the box: amplification ×20). **(C)** Paraffin sections of some xenograft tumors were immune-stained with several antibodies. **(D)** Schematic overview of Trop2 in the crosstalk with IGF2-IGF1R-Akt axis between cancer cells and TME in the gefitinib resistance of NSCLCs.

To assess the resistant response of NSCLC cell lines to an IGF1R inhibitor *in vivo*, we administered linsitinib to immunodeficient mice harboring tumors derived from PC-9/GR shNC and PC-9/GR shTrop2 cell lines companied with gefitinib treatment. After four weeks of linsitinib treatment, mice bearing PC-9/GR shTrop2 tumors showed a reduced level of tumor growth compared with other groups (Fig. 6A). We observed more necrotic tissue in tumors from PC-9/GR shTrop2 groups with linsitinib treatment than that in other groups (Fig. 6B). The nuclear membrane and some internal structures of the tumor cells were ruptured, and the chromosomal DNA in the nucleus was fragmented. We also analyzed whether infiltrating cells contribute to gefitinib drug resistance *in vivo*. We selected anti-CD45 antibody, anti-F4/80 antibody and anti-FSP-1 antibody to detect the expression of leukocyte, macrophage and fibroblasts. In drug resistance cell group with Trop2 knockdown and IGF1R inhibitor, there were more infiltrating cells between tumor cells (Fig. 6C). These results indicated that downregulation of Trop2 in drug resistant cells combined with an IGF1R inhibitor could recruit the inflammatory cells and remodeling the TME (Fig. 6D).

## Discussion

Trop2 is a transmembrane glycoprotein that is widely expressed on the surface of a variety of epithelial cell carcinoma cells and rarely expressed or not expressed in normal human tissues 24-26. Our previous research found that Trop2 induced epithelial‐mesenchymal transition through mediated β‐catenin in gastric cancer 18. Several targeted antibodies, antibody couplers and other forms of drugs targeting Trop2 have been developed 27. High expression of Trop2 can promote cell self-renewal and induce stem cell-like properties 17. Lin, et al. suggested that Trop2 plays an anti-cancer role due to epigenetic inactivation and inhibition of IGF1 signaling pathway in lung cancer 20. Another study reported that deletion of Trop2 in squamous cells promotes tumorigenesis and epithelial-mesenchymal transformation 21. In this study, we found no significant difference in the expression of Trop2 between NSCLC tumor tissues and paracancerous tissues, but the expression level of Trop2 was higher in NSCLC with EGFR mutation compared with those without mutation. Moreover, knocking down Trop2 inhibited cell proliferation and migration in gefitinib resistance in NSCLC cells (PC-9/GR) *in vitro*. Therefore, the specific role of Trop2 in lung cancer need to be further explored.

The IGF axis is regulated by complex interactions between ligands (IGF1 and IGF2), homologous receptors (IGF1R and IGF2R) and binding protein (IGFBPs). IGF2R belongs to type Ⅰ membrane integrin. IGF2R has a structural homology with IGF1R and high affinity to IGF2 and other ligands, but its affinity to IGF2 is greater than to IGF1 28. IGF2R exhibits anti-tumor effects *in vitro* and *in vivo*. IGF2R binds and degrades IGF2. IGF2 is highly expressed in a variety of tumor tissues, and activates the IGF1 signal pathway and promotes cancer progression on binding with IGF1R. When binding IGF2R, IGF2 is targeted for lysosomal degradation 29. In the study, a rescue experiment was designed to verify Trop2 bound IGF2R and activate the IGF2-IGF1R-Akt axis.

Our research simulated the human NSCLC TME *in vitro* and *in vivo* and demonstrated that Trop2 functions as a key player in modulating IGF2-IGF1R-Akt axis signaling for drug resistance in NSCLC and TME remodeling in NSCLC. Under co-culture conditions *in vitro*, macrophages and fibroblasts migrated towards gefitinib drug-resistant cell lines, and IGF2R was highly expressed in macrophages and fibroblast cells. The recruitment of macrophages and fibroblasts was inhibited by silencing Trop2 and was partly recovered by shIGF2R. Our *in vivo* experiments further indicated that shTrop2 in drug resistant cells with an IGF1R inhibitor could recruit infiltrating cells and remodel the TME.

TME is a dynamic network and a key factor affecting tumor metastasis, which may contribute to or hinder the immune response of anti-tumor cells 29-32. There can be a two-way interaction between tumor cells and interstitial cells in the TME. Tumor cells secrete growth factors and cytokines, and then recruit and regulate the interstitial cells and immune cells. In turn, the interaction between tumor cells and TME also affects the response of tumor cells to targeted therapy. The interaction between tumor cells and TME mainly promotes cell-to-cell adhesion 33. IGF1R has been regarded as one of the most promising targets for treating NSCLC. However, clinical studies confirmed that the overall response to treatment is not promising 16. Animal experiments showed that spontaneous metastasis of cancer cells occurred after a stable period of anti-IGF1R treatment in NSCLC tumor-bearing mice. Further research suggests that this response escape is not due to the failure of IGF1R blockade, but to the recruitment of macrophages, fibroblasts and cancer cells blocked by IGF1R. These cells are recruited into a large number of vascular endothelial cells aggregation, forming NSCLC TME, and different types of cells produced a good communication function, thus giving play to the mechanism of anti-tumor drug resistance 16. Therefore, drug resistance may be due not only to the excessive activity of tumor cells, but also to the complex interaction between tumor cells and heterogeneous stroma cells in the adjacent TME.

Current research indicated that a large number of factors produced by tumor cells promote the formation of the immunosuppressive TME by inhibiting the cytotoxicity of anti-tumor cells mediated by immune cells after gefitinib treatment in NSCLC patients. The TME is a dynamic network and a key factor affecting tumor metastasis.

Our research demonstrated the following results: (1) Trop2 up-regulated IGF2 level and promoted the development of gefitinib drug resistance in NSCLC by binding IGF2R; (2) Trop2 binding with IGF2R activated IGF2-IGF1R-Akt signaling to re-construct the TME and induced gefitinib resistance in NSCLC. These results provided molecular mechanistic insights into the crosstalk between cancer cells and the TME in NSCLC gefitinib resistance.

## Supplementary Material

Supplementary figures and tables.

## Figures and Tables

**Table 1:** Association between Trop2 expression and clinicopathologic characteristics in NSCLC patients

Characteristic	n	Trop2 expression		*P*	χ^2^
		Low or no	High		
**Age**				0.222	0.8552
<60	78	41 (52.60)	37 (47.40)		
≥60	86	39 (45.30)	47 (54.70)		
**Gender**				0.348	1.173
Male	85	38 (44.70)	47 (55.30)		
Female	79	42 (53.20)	37 (46.80)		
**Histological type**				0.559	0.094
Adenocarcinoma	84	41 (48.80)	43 (51.20)		
Squamous	80	38 (47.50)	42 (52.50)		
**Differentiation**				0.54	0.004
Poor	57	28 (49.10)	29 (50.90)		
Moderate-Well	107	52 (46.60)	55 (51.40)		
**TNM stage**				0.777	0.08
Ⅰ-Ⅱ	90	43 (47.80)	47 (52.20)		
Ⅲ-IV	74	37 (50.00)	37 (50.00)		
**Tumor size**				0.271	1.34
≤3 cm	95	50 (52.60)	45 (47.70)		
>3 cm	69	30 (43.50)	39 (56.50)		
**Lymph node metastases**				0.746	0.158
N0	59	30 (50.80)	29 (49.20)		
N1-3	105	50 (47.60)	55 (52.40)		
**Distant metastases**				0.54	0.949
M0	107	52 (48.60)	55 (51.40)		
M1	57	28 (49.10)	29 (50.90)		
**EGFR**				<0.001	56.592
Wild-type	86	66 (76.70)	20 (23.30)		
Mutant	78	14 (17.90)	64 (82.10)		
**Gefitinib valid**				0.027	5.068
≥9 months	58	40 (69.00)	18 (31.00)		
<9 months	25	23 (92.00)	2 (8.00)		

## Abbreviations

NSCLC: non-small cell lung cancer
TME: tumor microenvironment
Trop2: trophoblast cell surface antigen 2
IGF2R: insulin-like growth factor-2 receptor
RTK: receptor tyrosine kinases
