# Supplementary material for: Trop2 binding IGF2R induces gefitinib resistance in NSCLC by remodeling the tumor microenvironment
Source: J Cancer. 2021 Jul 3;12(17):5310–9. doi: 10.7150/jca.57711 (PMC8317539; doi:10.7150/jca.57711)
Supplement: Supplementary file 1 — Supplementary figures and tables. [file jcav12p5310s1.pdf]

Table S1 The expression levels of Trop2 in NSCLC

Characteristic	n	Trop2 expression		*P*	χ^2
		Low or no	high		
**Cancer tissues**	164	84(51.20)	80(48.80)	0.526	0.066
**Paracancerous tissues**	32	16(50.00)	16(50.00)		

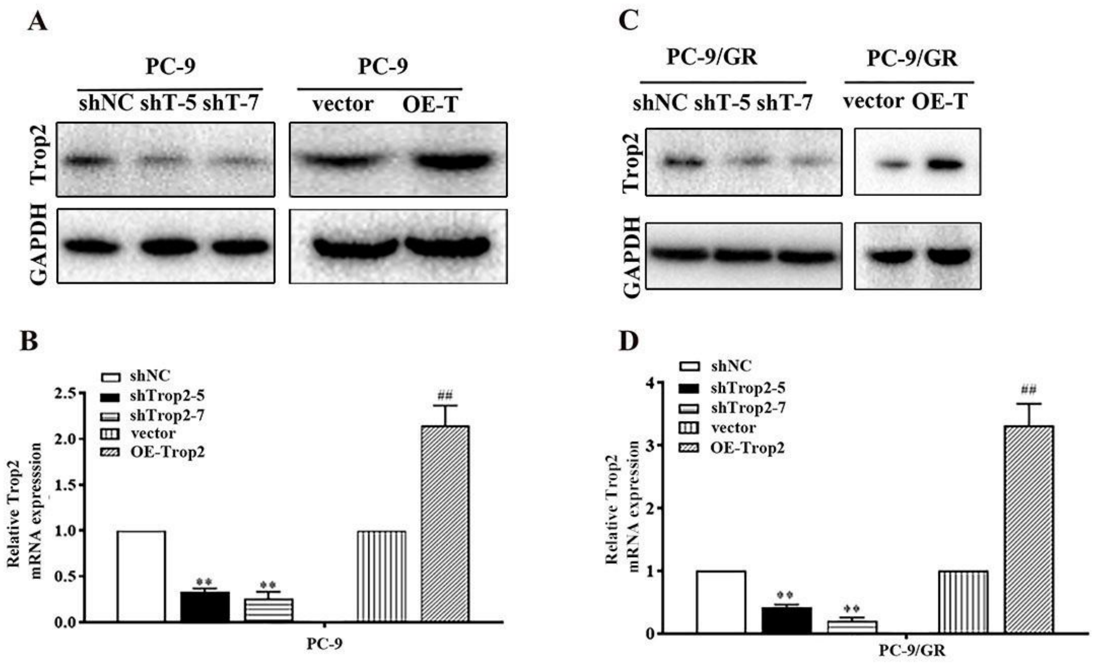

Figure S1. Knock-down and over-expression of Trop2 in PC-9 and PC-9/GR.

The efficiency of knock-down and over-expression Trop2 in PC-9 was detected by western blotting (A) and qRT-PCR (B). The efficiency of knock-down and over-expression Trop2 in PC-9/GR was detected by western blotting (C) and qRT-PCR (D). Mean ± SD, $^{**}P<0.01$, means compared with shNC group; $^{\#\#}P<0.001$, means compared with vector group.
